# dbMDEGA: a database for meta-analysis of differentially expressed genes in autism spectrum disorder

**DOI:** 10.1186/s12859-017-1915-2

**Published:** 2017-11-16

**Authors:** Shuyun Zhang, Libin Deng, Qiyue Jia, Shaoting Huang, Junwang Gu, Fankun Zhou, Meng Gao, Xinyi Sun, Chang Feng, Guangqin Fan

**Affiliations:** 1 Department of Occupational Health and Toxicology, School of Public Health, Nanchang University, BaYi Road 461, Nanchang, 330006 China; 2 Institute for Translational Medicine, Nanchang University, Nanchang, 330000 China; 3 Basic Medical College, Nanchang University, Nanchang, 330000 China; 4 Jiangxi Provincial Key Laboratory of Preventive Medicine, Nanchang University, Nanchang, 330006 China

**Keywords:** Gene expression, Meta-analysis, Database, Microarray

## Abstract

**Background:**

Autism spectrum disorders (ASD) are hereditary, heterogeneous and biologically complex neurodevelopmental disorders. Individual studies on gene expression in ASD cannot provide clear consensus conclusions. Therefore, a systematic review to synthesize the current findings from brain tissues and a search tool to share the meta-analysis results are urgently needed.

**Methods:**

Here, we conducted a meta-analysis of brain gene expression profiles in the current reported human ASD expression datasets (with 84 frozen male cortex samples, 17 female cortex samples, 32 cerebellum samples and 4 formalin fixed samples) and knock-out mouse ASD model expression datasets (with 80 collective brain samples). Then, we applied R language software and developed an interactive shared and updated database (dbMDEGA) displaying the results of meta-analysis of data from ASD studies regarding differentially expressed genes (*DEGs*) in the brain.

**Results:**

This database, dbMDEGA (https://dbmdega.shinyapps.io/dbMDEGA/), is a publicly available web-portal for manual annotation and visualization of *DEGs* in the brain from data from ASD studies. This database uniquely presents meta-analysis values and homologous forest plots of *DEGs* in brain tissues. Gene entries are annotated with meta-values, statistical values and forest plots of *DEGs* in brain samples. This database aims to provide searchable meta-analysis results based on the current reported brain gene expression datasets of ASD to help detect candidate genes underlying this disorder.

**Conclusion:**

This new analytical tool may provide valuable assistance in the discovery of *DEGs* and the elucidation of the molecular pathogenicity of ASD. This database model may be replicated to study other disorders.

**Electronic supplementary material:**

The online version of this article (10.1186/s12859-017-1915-2) contains supplementary material, which is available to authorized users.

## Background

Autism spectrum disorders (ASD) are clinically heterogeneous and biologically complex neurobehavioral disorders characterized by social communication deficits, impaired language development, repetitive activities and restrictive range of interests [1, 2]. In recent years, the incidence of autism has quickly increased; Lai et al. [3] have reported that the worldwide population prevalence is approximately 1%. Twin studies have suggested that genetic factors are important in the pathogenesis of ASD [3–5]; however, genes associated with ASD pathogenicity still need to be explored.

Microarray technology is a powerful tool used to provide evidence for the genetic contribution to ASD and other complex disorders [6–11]. In recent years, this technology has been applied to detect differentially expressed genes (*DEGs*) between autistic and normal individuals and to explore the pathology of ASD [6, 10–12]. For instance, Voineagu et al. [11] have further identified discrete modules of co-expressed genes associated with autism, such as the neuronal specific splicing factor *A2BP1*, and have provided evidence implicating transcriptional and splicing dysregulation as underlying mechanisms of neuronal dysfunction in ASD. Moreover, this technology has also been used on ASD mouse models and facilitates exploration of the possible molecular mechanisms of ASD [13, 14]. Finally, some studies have found significantly perturbed pathways in ASD, such as synaptic plasticity [13], neurogenesis and synaptic activity [12]. Collectively, these studies based on gene expression analysis can provide clues to guide future research.

Although microarray technology is a strategy to identify associated genes and underlying biological mechanisms, genes identified in one study often are not identified in other studies [15]. Combining information from multiple reported studies can also improve the reliability and generalizability of results [16]. Therefore, meta-analysis approaches have been used to identify consistent changes across multiple datasets and have already been successfully applied in different kinds of complex diseases [17–19]. For example, two meta-analyses of ASD [20, 21] have analyzed data from three human brain studies together with several blood studies and have identified some genes and pathways related to ASD with improved statistical power. Using RNA samples from either peripheral blood or brain tissue, these studies have identified many candidate genes such as *ATP5O, SLC25A12,* and *COX5B* [20]. However, they have mainly focused on mitochondrial [20] or ribosomal function [21], and currently, there is no potential solution for a customized query of meta-analysis results. To solve this problem, we built the database dbMDEGA, a new analytical tool that enables users to query for the statistical and meta-analysis values of a specific gene, and that provides reference datasets for exploring disease biology.

Moreover, another concern in ASD research relates to heterogeneity and tissue diversity such as the differences between blood and brain [19] and the differences among different regions of the brain [11]. For ASD studies, the advantage of using blood is that it is easier to collect from patients. However, blood may not be relevant to ASD or neurodevelopmental disorders, which presumably originate in the brain. Then, there may be constitutive differences in gene expression between the blood and brain [19, 22]. Voineagu et al. [11] have reported that gene expression changes associated with autism are more pronounced in the cortex. Here, to discover common *DEGs* in ASD with improved statistical power, we applied a systematic meta-analysis to three human brain gene expression datasets [6, 11, 23] with 84 collective frozen male cortex samples. Moreover, given our ability to visualize the diversity of different brain regions, states and sexes in people with autism compared with unaffected controls; we also collected 53 collective human brain samples (including 17 female cortex samples, 32 cerebellum samples and 4 formalin fixed samples) from three human brain gene expression datasets. Then, we established a database (dbMDEGA) including 17,742 human genes as meta-results for querying *DEGs* in ASD. Furthermore, to support discoveries in human studies, we also collected the current brain gene expression datasets for 14 ASD mouse models [24, 25] from 80 brain samples in five mouse datasets.

## Construction and content

### Data collection

We retrieved datasets from Gene Expression Omnibus (GEO) (http://www.ncbi.nlm.nih.gov/gds) by using the keyword “autism” on 3 May, 2015. Only expression profiles of brain tissue (cortex and cerebellum) from human ASD studies and mouse ASD models were used in further analysis (Tables 1, 2). Raw expression data generated by the providers for 3 human ASD studies (GEO accession numbers: GSE28475, GSE38322 and GSE28521) and 14 mouse models with ASD-related symptoms (GSE51612, GSE62594, GSE40630, GSE32012, and GSE47150; Table 3) were downloaded. Because the downloadable raw expression data for GSE28475 were already log2 transformed and normalized via quantile normalization with the *lumi* package in R language by the provider, to help ensure comparability and consistency, other raw expression datasets were independently preprocessed through background correction, log2 transformation and quantile normalization or Robust Multiarray Average implemented in the“*lumi* (for Illumina bead chip) [26]”, “*limma* (for Agilent bead chip) [27]” or “*affy* (for Affymetrix bead chip) [28]” R package as appropriate (Table 4). Moreover, the downloaded quantile normalization gene expression data for females and for fixed brain tissues in GSE28475 were also log2 transformed to ensure consistency with the meta-analysis data. To ensure comparability and consistency, we excluded 5 female cortex samples that did not meet the criteria (detected gene *p* < 0.05, outlier detection based on sample distance to “Center”, boxplot of microarray intensity) [6] of GSE28475 according to the reporter. The human brain sample information that was used in our database, after removal of duplicated samples, is shown in Additional file 1: Table S1 and Additional file 2: Table S2.

**Table 1:** Datasets of human brain used for Meta-Analysis

Data sets	Platform	Reference	Tissue type	Number of samples ASD;Control
Brain (male)				
GSE28475	GPL6883 (Illumina)	Chow et al. (2012)	Cortex	15;18
GSE28521	GPL6883 (Illumina)	Voineagu et al. (2011)	Frontal Cortex	9;14
GSE28521	GPL6883 (Illumina)	Voineagu et al. (2011)	temporal Cortex	7;11
GSE38322	GPL10558 (Illumina)	Ginsberg et al. (2012)	Occipital Cortex	4;6
				35;49 = 84
Brain (female)				
GSE28475	GPL6883 (Illumina)	Chow et al. (2012)	Cortex	5;4
GSE28521	GPL6883 (Illumina)	Voineagu et al. (2011)	Frontal Cortex	4;1
GSE28521	GPL6883 (Illumina)	Voineagu et al. (2011)	Temporal Cortex	3;1
Brain (other)				12;6 = 18
GSE28475	GPL6883 (Illumina)	Chow et al. (2012)	Formalin fixed Cortex	1;3
GSE28521	GPL6883 (Illumina)	Voineagu et al. (2011)	Cerebellum	5;11
GSE38322	GPL10558 (Illumina)	Ginsberg et al. (2012)	Cerebellum	8;8

**Table 2:** Datasets of mouse ASD model

Data sets	Platform	Reference	Tissue type	Number of samples ASD;Control
Brain				
GSE62594	GPL13912 (Agilent)	Shpyleva et al. (2014)	Cerebellum	8;8
GSE51612	GPL7202 (Agilent)	Sgadò et al. (2013)	Cerebellum	3;3
GSE40630	GPL6246 (Affymetrix)	Kong et al. (2014)	Cerebellum	8;8
GSE47150	GPL1261 (Affymetrix)	Lanz TA et al. (2013)	Cortex	30;4
GSE32012	GPL6246 (Affymetrix)	Horev G et al. (2011)	Cerebellum, Cortex	5;3
				54;26 = 80

**Table 3:** Mouse models of ASD in five datasets

Mouse model	Tissue type	Dataset	Experimental; Control
16p11.2(df/+)	Cortex	GSE32012	2;3
16p11.2(dp/+)	Cortex	GSE32012	2;3
MEF2D-KO	Cortex	GSE47150	3;4
NLGN1-KO	Cortex	GSE47150	4;4
PTEN-KO	Cortex	GSE47150	4;4
SHANK3-KO	Cortex	GSE47150	3;4
Fmr1-KO	Cortex	GSE47150	4;4
MeCP2-KO	Cortex	GSE47150	4;4
MEF2A-KO	Cortex	GSE47150	4;4
NLGN3-KO	Cortex	GSE47150	4;4
16p11.2(df/+)	Cerebellum	GSE32012	2;3
16p11.2(dp/+)	Cerebellum	GSE32012	3;3
Fmr1-KO	Cerebellum	GSE40630	5;5
Tsc2+/−	Cerebellum	GSE40630	3;3
En2−/−	Cerebellum	GSE51612	3;3
BTBR T + tf/J	Cerebellum	GSE62594	8;8

**Table 4:** Data processing of all gene expression datasets

Dataset	Chip Type	Data Processing	R Package
Human			
GSE28475	Illumina	Quantile normalization and log_2_ transformation	lumi
GSE28521	Illumina	Quantile normalization and log_2_ transformation	lumi
GSE28521	Illumina	Quantile normalization and log_2_ transformation	lumi
Mouse			
GSE62594	Agilent	Quantile normalization and log_2_ transformation	limma
GSE51612	Agilent	Quantile normalization and log_2_ transformation	limma
GSE40630	Affymetrix	RMA	affy
GSE47150	Affymetrix	RMA	affy
GSE32012	Affymetrix	RMA	affy

Mean gene expression values were computed for technical replicates to attain a single gene expression profile for each subject. We also conducted “Differential expression analysis” on each dataset by using *limma* R package [27] and obtained *p*-values for each probe between case and control. Probes that did not map to a gene were excluded. Then, all the *p-*values for each probe were ranked, for multiple probes that mapped to a gene, only probe with the lowest *p-*values was selected. All the gene expression datasets were corrected for batch effects with the *ComBat* function [29] of the R package *sva* [30]. Among all the datasets, the human studies contained 17,742 genes in common for meta-analysis, whereas in the mouse models, there were 12,109 genes in common with the genes in the human studies.

### Meta-analysis of gene expression data

Two meta-analysis methods were applied to the normalized male cortex sample expression data [31, 32]. These two methods that were applied to male cortex data were completed with the wrapper function of *metaMA* [32] in the R package *MAMA* [33]. In brief, the first approach (effect size combination method [32]) combines effect sizes from each dataset into a meta-effect size to estimate the amount of change in expression across all datasets. Datasets for each of the three human gene expression studies were generated from Illumina expression bead chips. The genes in common across studies were selected. Effect sizes for these unpaired datasets were calculated from moderated t-tests for each study, and then, these effect sizes were combined by using an explicitly random-effect model [32]. The result, denoted TestStatistic, is a vector with test statistics (“combined effect size”) in the meta-analysis. Then, according to the results of the test statistics, two-tailed *p-*values of the effect size combination method for each gene were computed, and Benjamini-Hochberg correction was used to correct the *p-*values for multiple hypothesis testing [34].

A second approach (*P*-value combination method) that combines *P*-values from individual experiments to identify genes with a large effect size in all datasets was also used. In the *P*-value combination method, *P*-values for these unpaired datasets were calculated from moderated t-tests for each study, and then, these *P*-values were combined by using an explicitly random-effect model [32]. The TestStatistic result is also a vector with test statistics (“combined *P*-values”) in meta-analysis. Then, according to the results of test statistics, two-tailed *p-*values of the effect size combination method for each gene were computed, and Benjamini-Hochberg correction was used to correct the *p-*values for multiple hypothesis testing [34].

Overall, *P*-value combination methods usually outperformed effect size combination approaches regarding sensitivity and gene ranking. Effect size combination methods were found to be more conservative. The ability of effect sizes to handle variance components was matched by *P*-value combination by using these moderated t-tests [32].

In addition, forest plots of the human brain samples and mouse brain samples were generated with the *metacont* function of the R package *meta* [35]. Random effects estimates for the meta-analyses were calculated with continuous outcome data, and the *p* value that was calculated in these forest plots described a heterogeneity test. For human brain samples, we applied the meta-analysis in the *metacont* function [35] to generate three forest plots that contained an only male cortex plot, an only cerebellum plot and separate cortex plot of the male cortex, female cortex and formalin cortex samples. For mouse model brain samples, we also applied the meta-analysis to generate three separate forest plots that contained only the cortex plot and two cerebellum plots of Affymetrix chip and Agilent chip.

### Design of database

After completion of the meta-analysis, the portal dbMDEGA was established in R language by using the *Shiny* R package [36], and it shows the calculated meta-analysis results of the genes, the corresponding forest plots and bean plots of the gene expression comparison between cases and controls. The bean plot visualizes univariate data between groups and shows data characteristics such as density curves, repeated observations and multimodal distribution. Users can access the established database online to obtain the corresponding meta-analysis results of this study.

### Database content

**Fig. 1:**
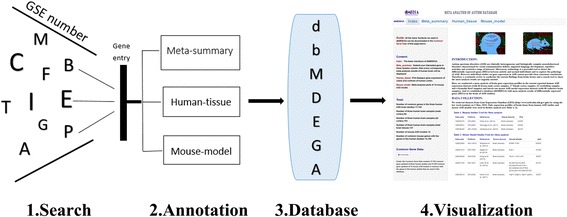
A flow diagram for the collection, annotation and presentation of associated genes for ASD. (1) The data in this database were obtained from our meta-analysis results and gene expression datasets of human and mouse ASD studies obtained from GEO DataSets (http://www.ncbi.nlm.nih.gov/gds). (2) Gene entry is organized for searching in the database. (3) The developed database is presented

The dbMDEGA was able to integrate ASD meta-analysis results from human brain tissues and mouse models and to display diverse annotations (Fig. 1). To help users and to ensure that they obtain the results for genes in this database, in the Common Gene Data of the Index sidebar panel, a downloadable file is provided containing all the common gene symbols used in the human studies and mouse ASD models. When a user clicks the “Download” mark below Common Gene Data, a common gene symbol file can be downloaded to the user’s computer. Here, the meta-analysis genes related to ASD, identified in three human studies (GSE28475, GSE28521, GSE38322), are annotated with three data panels: (i) In the Meta-summary panel, when users submit a gene, the unique meta-analysis results for the male cortex, determined through our calculations, are shown for each gene along with a forest plot showing the standardized mean difference in each of the three human ASD studies. For comparing the influence of brain regions, sex, and tissue state, this database provides an additional two separate forest plots (one for cerebellum samples and one for cortex samples, including female cortex and formalin cortex samples) to show the standardized mean difference in different parts of brain tissue and the different sexes and states. (ii) In the Human-tissue panel, statistical values of male cortex gene expression in people with ASD compared with normal individuals in each human study are displayed with a bean plot and a summary of mean, median and quartile values for cases and controls. (iii) In addition, we include a Mouse-model panel for comparison, which shows three separate forest plots (one for cortex samples and two cerebellum plots of Affymetrix chip and Agilent chip) of *DEGs* between mouse model and wild-type in 14 ASD models.

## Utility and discussion

### Search and display of dbMDEGA

**Fig. 2:**
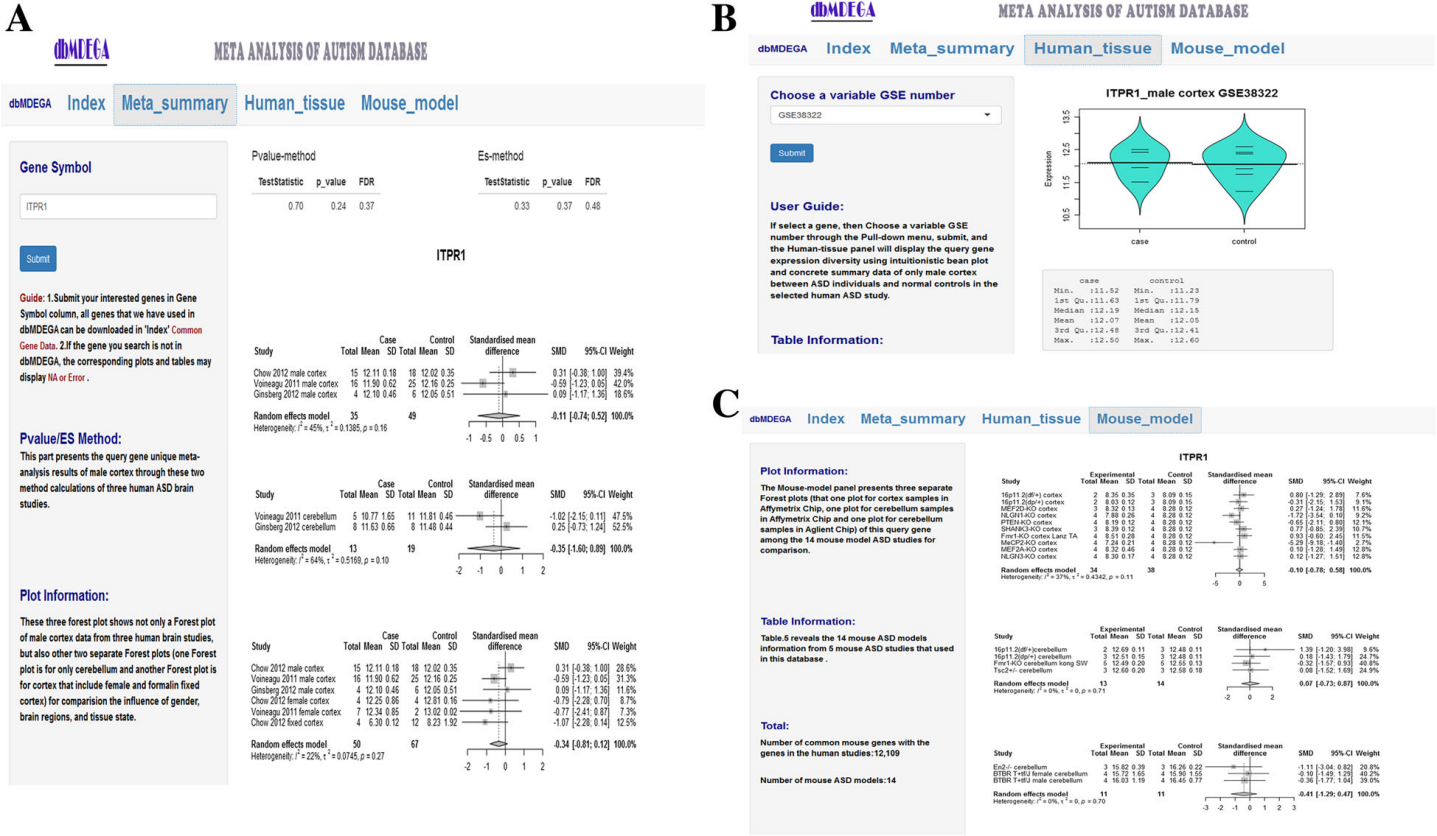
Online display of dbMDEGA search results. The example shows retrieval of a candidate gene, *ITPR1*, in dbMDEGA, (**a**) The meta-analysis results for male cortex together with three forest plots (for human male cortex samples; for human cerebellum samples; and for male, female and formalin fixed cortex) are displayed. **b** The statistical values of the candidate gene in one human dataset and a bean plot of the cases and controls are presented. **c** The candidate gene is also annotated with three forest plots of 14 mouse ASD model studies

User can click the “Download” button below Common Gene Data to download the common gene symbols we used in this database. The information in our database can be searched and visualized in several ways. A typical search result of our database is illustrated in Fig. 2. In this case, searching for a gene in the common gene symbols shows a list of information for this ASD-associated gene in the Web sidebar and main panel. This list contains the meta-analysis results, the candidate gene’s expression in different human studies, annotated with bean plot and summary results, and the results of mouse model studies, as shown in forest plots. The list shows the following: (i) In the Meta-summary panel, the user first inputs a gene symbol or gene name into the sidebar panel and submits the query. Then, the main panel reveals not only the values of effect size, *P*-value and false discovery rate (FDR) in the meta-analysis for this gene but also a forest plot of male cortex data from three human brain studies. Additionally, we provide two additional separate forest plots (one forest plot is for only the cerebellum and another forest plot is for cortex, including female and formalin fixed cortex) (Fig. 2a). (ii) In the Human-tissue panel, the user can select a GSE number from the human ASD studies (GSE28475, GSE38322, and GSE28521) and submit a query in the sidebar. The main panel displays the query gene’s expression diversity by using an intuitive bean plot of only the male cortex in ASD individuals and normal controls in the selected human ASD study. Additionally, concrete summary data of the gene’s expression in cases (human ASD) and controls (human non-ASD) is provided (Fig. 2b). (iii) The Mouse-model panel also presents three separate forest plots (one for cortex samples and two cerebellum plots of Affymetrix chip and Agilent chip) of the queried gene among the 14 mouse model ASD studies, for comparison (Fig. 2c). All the data in dbMDEGA are freely available for academic users. dbMDEGA can be accessed via (https://dbmdega.shinyapps.io/dbMDEGA/).

## Discussion

In our study, a meta-analysis was performed on current gene expression profiles of different brain tissues in human ASD studies and mouse ASD model studies; then, an open-access visualization database, dbMDEGA, was established with our meta-analysis results. dbMDEGA is the first database that displays the meta-analysis results of candidate DEGs in ASD, and it facilitates the exploration of unknown genetic causes of ASD. The corresponding results in the database are available for online searching, and may provide a reference for other researchers and follow-up studies. Furthermore, our database model could be replicated to study other disorders and establish corresponding databases of meta-analysis results.

Compared with other databases related to ASD (such as AutDB [37] and SFARI [24]), our database content is based on a systematic analysis of the existing gene expression datasets to indicate the overall differential expression of ASD candidate genes in different ASD studies. However, SFARI [24] and AutDB [37] both place emphasis on classifying and summarizing the candidate genes reported by published ASD studies. dbMDEGA can be used more intuitively to detect genetic causes of ASD. dbMDEGA can complement these two databases by providing systematic gene expression profile data on ASD, and it may help other researchers to further examine their genes of interest in ASD.

Genes contained in the visualization database dbMDEGA all have corresponding meta-analysis results and Forest plots together with bean plots, thus providing researchers with relatively more information that is intuitively understandable. For example, the reported neuronal specific splicing factor A2BP1, identified in previous ASD studies [10, 11], is statistically significant in dbMDEGA (TestStatistic = 2.73, *p*-value = 0.00, FDR = 0.07). In addition, compared with other existing meta-analysis reports, the visualization database dbMDEGA based on meta-analysis results has been consistent and inclusive. For instance, significant cellular respiration genes such as ATP5O (Meta *P*-value = 1.83 × 10–5), SLC25A12 (Meta *P*-value = 2.98 × 10–4), and COX5B (Meta *P*-value = 5.37 × 10–4) have been identified in other meta-analysis results [20]; in dbMDEGA, these genes also have a corresponding presentation (TestStatistic = 2.92, *p*-value = 0.00, FDR = 0.05; TestStatistic = 2.40, *p*-value = 0.01, FDR = 0.09; TestStatistic =2.11, *p*-value = 0.02, FDR = 0.13).

Heterogeneity between tissue samples and different studies is a considerable problem in expression profile analysis. Observations in diverse tissues such as the difference between blood and brain [19] and the difference among different regions of the brain [11] may be inconsistent and have not been fully explored in other meta-analysis studies of ASD. In our studies, only brain samples were used to perform the meta-analysis. For ASD studies, blood samples are easier to collect, but changes in the gene profile in the blood may not be observed in the brain, owing to tissue specificity [19, 22]. Hence, it is crucial to perform meta-analyses based on human brain samples for ASD studies.

Moreover, Voineagu et al. [11] have proposed gene expression differences between the cerebellum and cortex, and have indicated that gene expression changes associated with autism are more pronounced in the cerebral cortex. Ch’ng et al. [20] have also separated the cerebellum tissue and used the cortex of ASD cases and controls to conduct a meta-analysis. However, the verdict on gene expression changes between the cerebellum and cortex remains unclear. To intuitively show the difference among different regions of ASD in our database, we applied meta-analysis to obtain two separate forest plots: an only male cortex plot and an only cerebellum plot. Data from mouse models have been applied to the meta-analysis to obtain three forest plots that contain an only cortex plot and two cerebellum plots of Affymetrix chip and Agilent chip separately. To account for differences in sex and tissue state, we also applied the meta-analysis to generate one forest plot that contains a cortex plot of separate male cortex, female cortex and formalin cortex.

### Perspective

The occurrence of ASD, a severe neurodevelopmental disease, has increased significantly in recent years. Accumulating evidence suggests that genetic changes contribute to ASD, and studies reporting candidate genes associated with ASD are quickly accumulating. Here, we developed dbMDEGA to facilitate the discovery of candidate genes associated with ASD, on the basis of meta-analyses. In the future, when more ASD studies have been performed, we will update dbMDEGA accordingly.

## Conclusions

dbMDEGA is a publicly available web-portal and new analytical tool that allows for searchable meta-analysis results based on the current reported brain gene expression ASD datasets. This database is designed to share our meta-analysis results and provides valuable assistance in the discovery of *DEGs* and the molecular pathogenicity of ASD. Moreover, our database model could be replicated to study other disorders.

## Additional files

Additional file 1: Table S1. Brain samples of cortex included in the meta-analysis. (DOC 93 kb)

Additional file 2: Table S2. Brain samples of cerebellum included in the meta-analysis. (DOC 52 kb)

## Abbreviations

ASD: Autism spectrum disorders
DEGs: Differentially expressed genes
FDR: False discovery rate

## References

[1] Diagnostic and statistical manual of mental disorders DSM-5. Arlington, VA: American Psychiatric Association. 2015. http://dsm.psychiatryonline.org. Accessed 12 April 2015.

[2] De Rubeis S, Buxbaum JD (2015). Genetics and genomics of autism spectrum disorder: embracing complexity. Hum Mol Genet.

[3] Lai MC, Lombardo MV, Baron-Cohen S (2014). Autism. Lancet.

[4] Persico AM, Napolioni V (2013). Autism genetics. Behav Brain Res.

[5] Ronald A, Hoekstra RA (2011). Autism spectrum disorders and autistic traits: a decade of new twin studies. Am J Med Genet B Neuropsychiatr Genet.

[6] Chow ML, Winn ME, Li HR, April C, Wynshaw-Boris A, Fan JB, XD F, Courchesne E, Schork NJ (2012). Preprocessing and quality control strategies for Illumina DASL assay-based brain gene expression studies with semi-degraded samples. Front Genet.

[7] Bunney WE, Bunney BG, Vawter MP, Tomita H, Li J, Evans SJ, Choudary PV, Myers RM, Jones EG, Watson SJ, Akil H (2003). Microarray technology: a review of new strategies to discover candidate vulnerability genes in psychiatric disorders. Am J Psychiatry.

[8] DeRisi J, Penland L, Brown PO, Bittner ML, Meltzer PS, Ray M, Chen Y, YA S, Trent JM (1996). Use of a cDNA microarray to analyse gene expression patterns in human cancer. Nat Genet.

[9] Mehta D, Menke A, Binder EB (2010). Gene expression studies in major depression. Curr Psychiatry Rep.

[10] Sarachana T, Hu VW (2013). Genome-wide identification of transcriptional targets of RORA reveals direct regulation of multiple genes associated with autism spectrum disorder. Mol Autism..

[11] Voineagu I, Wang X, Johnston P, Lowe JK, Tian Y, Horvath S, Mill J, Cantor RM, Blencowe BJ, Geschwind DH (2011). Transcriptomic analysis of autistic brain reveals convergent molecular pathology. Nature.

[12] Lanz TA, Guilmette E, Gosink MM, Fischer JE, Fitzgerald LW, Stephenson DT, Pletcher MT (2013). Transcriptomic analysis of genetically defined autism candidate genes reveals common mechanisms of action. Mol Autism..

[13] Kong SW, Sahin M, Collins CD, Wertz MH, Campbell MG, Leech JD, Krueger D, Bear MF, Kunkel LM, Kohane IS (2014). Divergent dysregulation of gene expression in murine models of fragile X syndrome and tuberous sclerosis. Mol Autism.

[14] Shpyleva S, Ivanovsky S, de Conti A, Melnyk S, Tryndyak V, Beland FA, James SJ, Pogribny IP, Cerebellar Oxidative DNA (2014). Damage and altered DNA Methylation in the BTBRT+tf/J mouse model of autism and similarities with human post mortem cerebellum. PLoS One.

[15] Elashoff M, Higgs BW, Yolken RH, Knable MB, Weis S, Webster MJ, Barci BM, Torrey EF (2007). Meta-analysis of 12 genomic studies in bipolar disorder. J Mol Neurosci.

[16] Ramasamy A, Mondry A, Holmes CC, Altman DG (2008). Key issues in conducting a meta-analysis of gene expression microarray datasets. PLoS Med.

[17] Chen R, Khatri P, Mazur PK, Polin M, Zheng Y, Vaka D, Hoang CD, Shrager J, Xu Y, Vicent S, Butte AJ, Sweet-Cordero EAA (2014). Meta-analysis of lung cancer gene expression identifies PTK7 as a survival gene in lung adenocarcinoma. Cancer Res.

[18] Santiago JA, Potashkin JA (2015). Network-based meta-analysis identifies HNF4A and PTBP1 as longitudinally dynamic biomarkers for Parkinson's disease. Proc Natl Acad Sci U S A.

[19] Seifuddin F, Pirooznia M, Judy JT, Goes FS, Potash JB, Zandi PP (2013). Systematic review of genome-wide gene expression studies of bipolar disorder. BMC Psychiatry.

[20] Ch’ng C, Kwok W, Rogic S, Pavlidis P (2015). Meta-analysis of gene expression in autism Spectrum disorder. Autism Res.

[21] Ning LF, YQ Y, GuoJi ET, Kou CG, YH W, Shi JP, Ai LZ, Yu Q (2015). Meta-analysis of differentially expressed genes in autism based on gene expression data. Genet Mol Res.

[22] Leonard S, Logel J, Luthman D, Casanova M, Kirch D, Freedman R (1993). Biological stability of mRNA isolated from human postmortem brain collections. Biol Psychiatry.

[23] Ginsberg MR, Rubin RA, Falcone T, Ting AH, Natowicz MR (2012). Brain transcriptional and epigenetic associations with autism. PLoS One.

[24] Banerjee-Basu S, Packer A (2010). SFARI gene: an evolving database for the autism research community. Dis Model Mech.

[25] Ellegood J, Anagnostou E, Babineau BA, Crawley JN, Lin L, Genestine M, DiCicco-Bloom E (2015). Clustering autism: using neuroanatomical differences in 26 mouse models to gain insight into the heterogeneity. Mol Psychiatry.

[26] Du P, Kibbe WA, Lin SM (2008). lumi: a pipeline for processing Illumina microarray. Bioinformatics.

[27] Ritchie ME, Phipson B, Wu D, Hu Y, Law CW, Shi W, Smyth GK (2015). Limma powers differential expression analyses for RNA-sequencing and microarray studies. Nucleic Acids Res.

[28] Gautier L, Cope L, Bolstad BM, Irizarry RA (2004). Affy---analysis of Affymetrix GeneChip data at the probe level. Bioinformatics.

[29] Johnson WE, Li C, Rabinovic A (2007). Adjusting batch effects in microarray expression data using empirical Bayes methods. Biostatistics.

[30] Jeffrey T, Leek W, Johnson E, Parker HS, Fertig EJ, Jaffe AE, John D (2015). Storey. Sva: surrogate variable analysis.

[31] Khatri P, Roedder S, Kimura N, De Vusser K, Morgan AA, Gong Y, Fischbein MP, Robbins RC, Naesens M, Butte AJ, Sarwal MM (2013). A common rejection module (CRM) for acute rejection across multiple organs identifies novel therapeutics for organ transplantation. J Exp Med.

[32] Marot G, Foulley JL, Mayer CD, Jaffrézic F (2009). Moderated effect size and P-value combinations for microarray meta-analyses. Bioinformatics.

[33] Ivana I (2013). MAMA: meta-analysis of MicroArray.

[34] Benjamini Y, Hochberg Y (1995). Controlling the false discovery rate: a practical and powerful approach to multiple testing. J R Stat Soc Ser B.

[35] Guido S (2007). Meta: an R package for meta-analysis. R News.

[36] Winston C, Joe C, Allaire JJ, Yihui X, Jonathan M (2017). Shiny: web application framework for R.

[37] Basu SN, Kollu R, Banerjee-Basu S (2009). AutDB: a gene reference resource for autism research. Nucleic Acids Res.

