# Supplementary material for: dbMDEGA: a database for meta-analysis of differentially expressed genes in autism spectrum disorder
Source: BMC Bioinformatics. 2017 Nov 16;18:494. doi: 10.1186/s12859-017-1915-2 (PMC5691387; doi:10.1186/s12859-017-1915-2)
Supplement: Supplementary file 1 — Brain samples of cortex included in the meta-analysis. (DOC 93 kb) [file 12859_2017_1915_MOESM1_ESM.doc]

**Additional file 1: Table S1**. Brain samples of cortex included in the meta-analysis.

| Date Set | Sample ID | Gender | Brain Region | Disease Status | Preservation |
| --- | --- | --- | --- | --- | --- |
| GSE38322 | AN03345 | M | cortex | autism | Frozen |
| GSE38322 | AN06420 | M | cortex | autism | Frozen |
| GSE38322 | AN08873 | M | cortex | autism | Frozen |
| GSE38322 | AN10723 | M | cortex | Control | Frozen |
| GSE38322 | AN10833 | M | cortex | Control | Frozen |
| GSE38322 | AN19511 | M | cortex | autism | Frozen |
| GSE38322 | BTB1453 | M | cortex | Control | Frozen |
| GSE38322 | BTB3228 | M | cortex | Control | Frozen |
| GSE38322 | UMB4543 | M | cortex | Control | Frozen |
| GSE38322 | UMB4670 | M | cortex | Control | Frozen |
| GSE28521 | AN00142 | M | cortex | control | Frozen |
| GSE28521 | AN00493 | M | cortex | autism | Frozen |
| GSE28521 | AN00544 | M | cortex | control | Frozen |
| GSE28521 | AN00764 | M | cortex | autism | Frozen |
| GSE28521 | AN01125 | M | cortex | control | Frozen |
| GSE28521 | AN01410 | M | cortex | control | Frozen |
| GSE28521 | AN03217 | M | cortex | control | Frozen |
| GSE28521 | AN04479 | M | cortex | control | Frozen |
|  |  |  |  |  | Continued… |
| Date Set | Sample ID | Gender | Brain Region | Disease Status | Preservation |
| GSE28521 | AN06420 | M | cortex | autism | Frozen |
| GSE28521 | AN07176 | M | cortex | control | Frozen |
| GSE28521 | AN08166 | M | cortex | autism | Frozen |
| GSE28521 | AN08792 | M | cortex | autism | Frozen |
| GSE28521 | AN10028 | M | cortex | control | Frozen |
| GSE28521 | AN11989 | M | cortex | autism | Frozen |
| GSE28521 | AN12137 | M | cortex | control | Frozen |
| GSE28521 | AN12240 | M | cortex | control | Frozen |
| GSE28521 | AN14757 | M | cortex | control | Frozen |
| GSE28521 | AN16641 | M | cortex | autism | Frozen |
| GSE28521 | AN17138 | M | cortex | autism | Frozen |
| GSE28521 | AN17254 | M | cortex | autism | Frozen |
| GSE28521 | AN17425 | M | cortex | control | Frozen |
| GSE28521 | AN19442 | M | cortex | control | Frozen |
| GSE28521 | AN19760 | M | cortex | control | Frozen |
| GSE28475 | B1469 | M | cortex | Autism | Frozen |
| GSE28475 | B4756 | M | cortex | Control | Frozen |
| GSE28475 | B4925 | M | cortex | Autism | Frozen |
| GSE28475 | B5173 | M | cortex | Autism | Frozen |
|  |  |  |  |  | Continued… |
| Date Set | Sample ID | Gender | Brain Region | Disease Status | Preservation |
| GSE28475 | B5251 | M | cortex | Control | Frozen |
| GSE28475 | B5334 | M | cortex | Control | Frozen |
| GSE28475 | B5352 | M | cortex | Control | Frozen |
| GSE28475 | B5666 | M | cortex | Autism | Frozen |
| GSE28475 | B5813 | M | cortex | Control | Frozen |
| GSE28475 | B5873 | M | cortex | Control | Frozen |
| GSE28475 | B6399 | M | cortex | Autism | Frozen |
| GSE28475 | B6677 | M | cortex | Autism | Frozen |
| GSE28475 | B6756 | M | cortex | Control | Frozen |
| GSE28475 | B6860 | M | cortex | Control | Frozen |
| GSE28475 | B6994 | M | cortex | Autism | Frozen |
| GSE28475 | B7079 | M | cortex | Autism | Frozen |
| GSE28475 | B7109 | M | cortex | Autism | Frozen |
| GSE28475 | UMB1349 | M | cortex | Autism | Frozen |
| GSE28475 | UMB1445 | M | cortex | Autism | Frozen |
| GSE28475 | UMB1500 | M | cortex | Control | Frozen |
| GSE28475 | UMB1649 | M | cortex | Control | Frozen |
| GSE28475 | UMB1650 | M | cortex | Control | Frozen |
| GSE28475 | UMB1674 | M | cortex | Control | Frozen |
|  |  |  |  |  | Continued… |
| Date Set | Sample ID | Gender | Brain Region | Disease Status | Preservation |
| GSE28475 | UMB1714 | M | cortex | Control | Frozen |
| GSE28475 | UMB1796 | M | cortex | Control | Frozen |
| GSE28475 | UMB4231 | M | cortex | Autism | Frozen |
| GSE28475 | UMB4722 | M | cortex | Control | Frozen |
| GSE28475 | UMB4787 | M | cortex | Control | Frozen |
| GSE28475 | UMB4849 | M | cortex | Autism | Frozen |
| GSE28475 | UMB4898 | M | cortex | Control | Frozen |
| GSE28475 | UMB4899 | M | cortex | Autism | Frozen |
| GSE28475 | UMB797 | M | cortex | Autism | Frozen |
| GSE28475 | UMB818 | M | cortex | Control | Frozen |
| GSE28521 | AN01570 | F | cortex | autism | Frozen |
| GSE28521 | AN12457 | F | cortex | autism | Frozen |
| GSE28521 | AN16115 | F | cortex | autism | Frozen |
| GSE28521 | AN17777 | F | cortex | autism | Frozen |
| GSE28521 | AN15566 | F | cortex | control | Frozen |
| GSE28475 | UMB1499 | F | cortex | Control | Frozen |
| GSE28475 | UMB1407 | F | cortex | Control | Frozen |
| GSE28475 | UMB1377 | F | cortex | Control | Frozen |
| GSE28475 | B6736 | F | cortex | Control | Frozen |
|  |  |  |  |  | Continued… |
| Date Set | Sample ID | Gender | Brain Region | Disease Status | Preservation |
| GSE28475 | UMB1174 | F | cortex | Autism | Frozen |
| GSE28475 | B7085 | F | cortex | Autism | Frozen |
| GSE28475 | B6184 | F | cortex | Autism | Frozen |
| GSE28475 | UMB4671 | F | cortex | Autism | Frozen |
| GSE28475 | BTB3859 | M | cortex | control | Formalin Fixed |
| GSE28475 | UMB1670 | M | cortex | control | Formalin Fixed |
| GSE28475 | UMB1796 | M | cortex | control | Formalin Fixed |
| GSE28475 | B5223 | M | cortex | autism | Formalin Fixed |
|  |  |  |  |  |  |

**Title:** dbMDEGA: a database for meta-analysis of differentially expressed genes in Autism Spectrum Disorder

**Journal name:** BMC Bioinformatics

**Author name:** Shuyun Zhang^1, 3^, Libin Deng^2^, Qiyue Jia^1^, Shaoting Huang^1^, Junwang Gu^1^, Fankun Zhou^1^, Meng Gao^2^, Xinyi Sun^2^, Chang Feng^1^, Guangqin Fan^1, 3^*

**Affiliation:** 1 Department of Occupational Health and Toxicology, School of Public Health,

Nanchang University, BaYi Road 461, Nanchang 330006, P. R. China.

2 Institute for Translational Medicine, Nanchang University, Nanchang 330000, China; Basic Medical College, Nanchang University, Nanchang 330000, China.

3 Jiangxi Provincial Key Laboratory of Preventive Medicine, Nanchang University, Nanchang 330006, P.R. China

***Corresponding author** **E-mail:** fanguangqin@ncu.edu.cn
