# Supplementary material for: dbMDEGA: a database for meta-analysis of differentially expressed genes in autism spectrum disorder
Source: BMC Bioinformatics. 2017 Nov 16;18:494. doi: 10.1186/s12859-017-1915-2 (PMC5691387; doi:10.1186/s12859-017-1915-2)
Supplement: Supplementary file 2 — Brain samples of cerebellum included in the meta-analysis. (DOC 52 kb) [file 12859_2017_1915_MOESM2_ESM.doc]

Additional file 2: Table S2. Brain samples of cerebellum included in the meta-analysis.

| Date Set | Sample ID | Gender | Brain Region | Disease Status | Preservation |
| --- | --- | --- | --- | --- | --- |
| GSE28521 | AN16115 | M | cerebellum | autism | Frozen |
| GSE28521 | AN16641 | M | cerebellum | autism | Frozen |
| GSE28521 | AN17254 | M | cerebellum | autism | Frozen |
| GSE28521 | AN01570 | M | cerebellum | autism | Frozen |
| GSE28521 | AN00764 | M | cerebellum | autism | Frozen |
| GSE28521 | AN10028 | M | cerebellum | controls | Frozen |
| GSE28521 | AN12240 | M | cerebellum | controls | Frozen |
| GSE28521 | AN13295 | M | cerebellum | controls | Frozen |
| GSE28521 | AN14757 | M | cerebellum | controls | Frozen |
| GSE28521 | AN15566 | F | cerebellum | controls | Frozen |
| GSE28521 | AN17425 | M | cerebellum | controls | Frozen |
| GSE28521 | AN19442 | M | cerebellum | controls | Frozen |
| GSE28521 | AN19760 | M | cerebellum | controls | Frozen |
| GSE28521 | AN01125 | M | cerebellum | controls | Frozen |
| GSE28521 | AN03217 | M | cerebellum | controls | Frozen |
| GSE28521 | AN00142 | M | cerebellum | controls | Frozen |
| GSE38322 | AN03345 | M | cerebellum | autism | Frozen |
| GSE38322 | AN06420 | M | cerebellum | autism | Frozen |
| GSE38322 | AN08166 | M | cerebellum | autism | Frozen |
| GSE38322 | AN08873 | M | cerebellum | autism | Frozen |
| GSE38322 | AN09730 | M | cerebellum | autism | Frozen |
| GSE38322 | AN11989 | M | cerebellum | autism | Frozen |
|  |  |  |  |  | Continued… |
| Date Set | Sample ID | Gender | Brain Region | Disease Status | Preservation |
| GSE38322 | AN17678 | M | cerebellum | autism | Frozen |
| GSE38322 | AN19511 | M | cerebellum | autism | Frozen |
| GSE38322 | AN05475 | M | cerebellum | control | Frozen |
| GSE38322 | AN10723 | M | cerebellum | control | Frozen |
| GSE38322 | AN10833 | M | cerebellum | control | Frozen |
| GSE38322 | AN15622 | M | cerebellum | control | Frozen |
| GSE38322 | BTB1453 | M | cerebellum | control | Frozen |
| GSE38322 | BTB3228 | M | cerebellum | control | Frozen |
| GSE38322 | UMB4543 | M | cerebellum | control | Frozen |
| GSE38322 | UMB4670 | M | cerebellum | control | Frozen |
|  |  |  |  |  |  |

Title: dbMDEGA: a database for meta-analysis of differentially expressed genes in Autism Spectrum Disorder

Journal name: BMC Bioinformatics

Author name: Shuyun Zhang^1, 3^, Libin Deng^2^, Qiyue Jia^1^, Shaoting Huang^1^, Junwang Gu^1^, Fankun Zhou^1^, Meng Gao^2^, Xinyi Sun^2^, Chang Feng^1^, Guangqin Fan^1,3^*

Affiliation: 1 Department of Occupational Health and Toxicology, School of Public Health,

Nanchang University, BaYi Road 461, Nanchang 330006, P. R. China.

2 Institute for Translational Medicine, Nanchang University, Nanchang 330000, China; Basic Medical College, Nanchang University, Nanchang 330000, China.

3 Jiangxi Provincial Key Laboratory of Preventive Medicine, Nanchang University, Nanchang 330006, P.R. China

*Corresponding author E-mail: fanguangqin@ncu.edu.cn
